# Physicochemical and Volatile Flavor Properties of Fish Skin under Conventional Frying, Air Frying and Vacuum Frying

**DOI:** 10.3390/molecules28114376

**Published:** 2023-05-26

**Authors:** Ming-Chih Fang, Peng-Shih-Yun Chin, Wen-Chieh Sung, Tai-Yuan Chen

**Affiliations:** 1 Department of Food Science, National Taiwan Ocean University, Keelung 202301, Taiwan; mcfang@mail.ntou.edu.tw (M.-C.F.); milk759759@gmail.com (P.-S.-Y.C.); sungwill@mail.ntou.edu.tw (W.-C.S.); 2 Center of Excellence for the Oceans, National Taiwan Ocean University, Keelung 202301, Taiwan

**Keywords:** air frying, by-products, circular economy, lipid oxidation, tilapia skin, vacuum frying, volatile compounds

## Abstract

The aim of this study was to investigate the physicochemical characteristics and volatile flavor of fried tilapia skins under three frying methods. Conventional deep-fat frying usually increases the oil content of the fried fish skin and leads to lipid oxidation, which reduces the product quality. Alternative frying methods, such as air frying for 6 and 12 min under 180 °C (AF6, and AF12) and vacuum frying at 0.085 MPa for 8 and 24 min under 120 °C (VF8, and VF24) were compared to conventional frying for 2 and 8 min under 180 °C (CF2, and CF8) for tilapia skin. Physical properties of fried skin, such as the moisture content, water activity, L* values and breaking force decreased under all frying methods, while the lipid oxidation and a*, b* values increased with the increase in frying time. In general, VF offered higher hardness of product compared to AF which had a lower breaking force. Especially AF12 and CF8 had the lowest breaking force, which indicated higher crispness. For the oil quality inside the product, AF and VF reduced conjugated dienes formation and retarded oxidation compared to CF. The results of the flavor compositions of fish skin measured using gas chromatography mass spectrometry (GC/MS) with solid phase microextraction (SPME) showed that CF obtained higher unpleasant oily odor (nonanal, 2,4-decadienal, etc.), while AF presented greater grilling flavor (pyrazine derivatives). Because fish skin fried by AF only relied on hot air, Maillard reaction derived compounds, such as methylpyrazine, 2,5-dimethylpyrazine, and benzaldehyde were the leading flavors. This made the aroma profiles of AF very different from VF and CF. Among all the approaches, AF and VF developed lower oil content, mild fat oxidation and better flavor attributes, which proves their practical applications for frying tilapia fish skin.

## 1. Introduction

Tilapia is an important fish species and combined with other cichlids are the second-largest produced aquatic products, accounting between 0.7 and 0.9 million tonnes per year worldwide [1]. In Taiwan, there are about 4500 tonnes of tilapia production each year. However, 63.9% of its entire content are by-products, such as heads, bones, skins and scales, which are always discarded. Finding alternative ways to utilize these by-products and transforming them into value-added products has been a major challenge for the food processing technology and the sustainable food value chains (SFVC) [2]. One popular way of processing fish by-products is making crispy fish skin snack; this dish has recently garnered widespread attraction of consumers, especially in Asian countries, such as Singapore, Thailand, China, Philippines, and Taiwan.

Conventional frying (deep-fat frying, CF) is a process through which food materials are either completely or partially immersed in hot oil, typically between 165 °C and 190 °C. During CF, heat is transferred from oil to the food materials, which rapidly increases the surface temperature of the food and simultaneously water in fried products evaporates [3]. Frying provides delectable foods with a golden color and unique sensory characteristics. The process is simple, convenient, and commonly used for preparing fish by consumers [4]. Although there are several advantages of frying, some adverse effects are commonly observed, such as excessive darkening and scorching of the products, especially in seafoods rich in polyunsaturated fatty acids (PUFAs) and phospholipids. Other unfavorable effects include increased fat/oil intake content [5], changes in fatty acid profile [6], and generation of oxidized and polymerized lipid products [7], which are responsible for undesirable sensory, odor, and quality [6,7]. Alternative frying techniques, such as air frying (AF) and vacuum frying (VF) apply low quantities of oil/fat for frying which leads to less oil absorption and lipid oxidation, while maintaining the nutritional quality, color, odor and other textural attributes compared to conventional frying. AF greatly reduces the use of frying oil, and effectively completes cooking through hot air and oil droplets spray around the raw food materials. Hence, it results in very low oil absorption on the surface of fried foods. VF fries foods under pressures well below the atmospheric levels in order to lower the boiling point of water, thereby accelerating the cooking process and preventing chemical degradative reactions, such as oxidation and enzymatic browning [8]. Although different frying methods used in seafoods processing had been reported, there were only a few studies focusing on the volatile flavor properties of fried aquatic by-products [9,10,11]. The volatile odor compounds (VOCs) played an important role in determining the sensory quality, consumer’s choice and the intake of a fried food. These VOCs were generally generated via thermal-induced Maillard reaction, Strecker and lipid degradation. The VOCs including aldehydes, ketones, alcohols, esters, pyrazines, hydrocarbons, acids, amines and other heterocyclic compounds were commonly distributed and tested for key flavor characteristics in fried aquatic foods [10,11]. AF fried fish skin was found to exhibit higher VOCs and consumer acceptance than fish skin cooked with CF, rotary baking and infrared radiation [10].

Previously, our group reported that based on mass transfer and texture issues, air frying and vacuum frying were good alternatives to conventional frying of fried fish skin [9]. Recently, CF, AF, rotary baking and infrared radiation were used to prepare fried tilapia skin snacks [10], and key aroma compounds of fried tilapia in soybean oil was also investigated [11]. However, the concrete oxidation, browning and volatile flavors of fried fish skin under air frying, vacuum frying and conventional deep-fat frying were still lacking. Therefore, the objective of this current study was to analyze the changes in physicochemical attributes including moisture content, water activity, breaking force, color, oil absorption, lipid oxidation (conjugated diene value, CDV and the thiobarbituric acid reactive substances, TBARS), and VOCs under six frying conditions. The tilapia skin was further divided into two parts: the belly (BE) and the back (BK). BE contained the light muscle and was abundant in neutral lipids, while BK contained the dark muscle and was abundant in phospholipid, PUFAs, haemopigments, and myoglobin. The comparison among the frying methods and the effects observed on the light and dark muscle of fried tilapia skin were then discussed in this study. The correlation among frying methods, frying temperature/time, light muscle and dark muscle as well as VOCs of fried tilapia skin were finally classified and visualized using principal component analysis (PCA).

## 2. Results and Discussion

### 2.1. Physicochemical Attributes

Frying conditions, such as conventional frying for 2 and 8 min under 180 °C (CF2, and CF8), air frying for 6 and 12 min under 180 °C (AF6, and AF12), and vacuum frying at 0.085 MPa for 8 and 24 min under 120 °C (VF8, and VF24) (Table 1) were referred in our previous study on mass transfer, moisture, water activity and breaking force parameters of fried fish skin [9]. The thermal processing at 180 °C was generally regarded as the ideal frying temperature point that produces unique satisfying flavor and enhances the quality of food. Thus, frying temperature below or above 180 °C might cause adverse effects. In French fries, when the frying temperature was reduced from 180 °C to 120 °C, it led to a 32% increase in oil absorption [12]. In AF, the lowest oil absorption and breaking force demonstrating optimal frying temperature was 120 °C at 0.085 MPa for French fries [9,13]. Acrylamide contents in French fries were reported to increase by 144% when the frying temperature was increased from 180 °C to 190 °C [14]. Additionally, innovative UV-C radiation contributed to an increase in acrylamide levels of French fries but soaking of semi-products in water also reduced its contents [15]. The N^ε^-carboxymethyllysine and benzo(a)pyrene were the main food hazard factors in the direct fried fish. On the other hand, acrylamide was the major hazard factor in the flour-coated fried fish [16].

**Figure 1:**
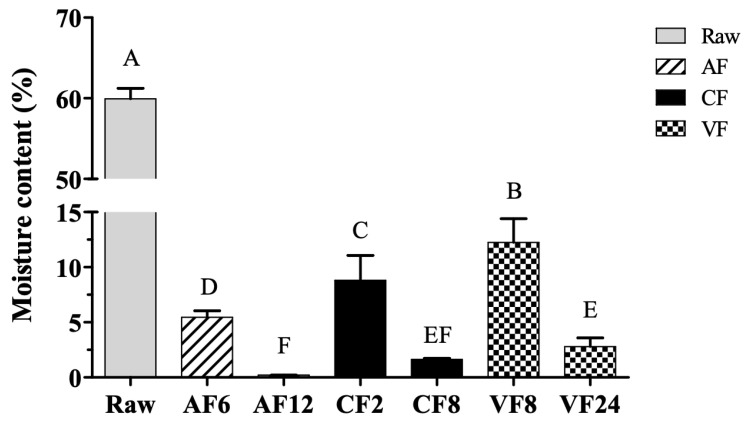
Changes in moisture content of fried tilapia skin with different frying methods. Different letter for any given treatment are significantly different (*n* = 3, *p* < 0.05). Abbreviation: AF6 & AF12 (Air frying at 6 and 12 min); CF2 & CF8 (Conventional frying at 2 and 8 min); VF8 & VF24 (Vacuum frying at 8 and 24 min).

Moisture content is an important quality attribute, and it was negatively correlated with crispy surface and shelf life of fried foods. The moisture content of raw tilapia skin was initially 59.93% and reduced with the increase in frying temperature and extension of time (Figure 1). The highest moisture content was found in VF8 (12.25%). The lowest moisture content was found in AF12 (0.21%); no significant difference in moisture content was observed with CF8. VF required to prolong the frying time for a considerable time than AF and CF as its cooking temperature was rather low. The lower frying temperature (120 °C) in VF slowed the rate of mass water transfer which resulted in high moisture content of the food cooked in VF [9].

**Figure 2:**
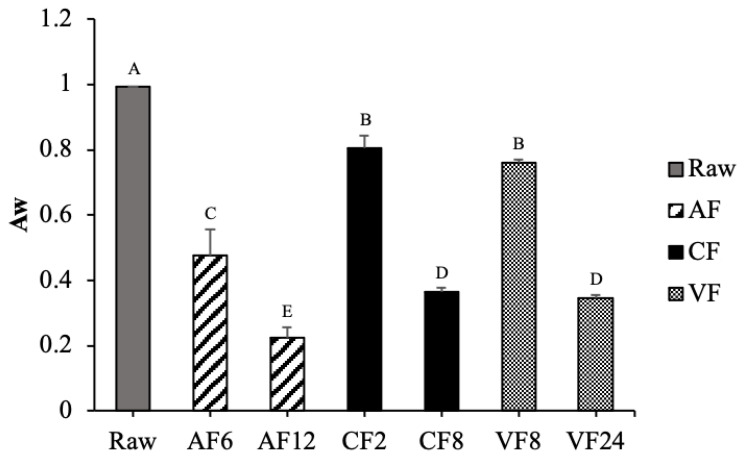
Changes in water activity of fried tilapia skin with different frying methods. Different letter for any given treatment are significantly different (*n* = 3, *p* < 0.05). Abbreviation: AF6 & AF12 (Air frying at 6 and 12 min); CF2 & CF8 (Conventional frying at 2 and 8 min); VF8 & VF24 (Vacuum frying at 8 and 24 min).

Water activity is a characteristic indicator for evaluating the safety and stability of the shelf life of food. The initial water activity of raw tilapia skin was 0.994 (Figure 2). The lowest water activity was observed in AF12 (0.225), which is in alignment with the above results of moisture content. CF and VF showed similar trends of declining water activities. However, VF required more time (24 min) to reach the same level of water activity compared to CF at 8 min.

Color value represents the appearance of fried food which has a great influence on the purchasing intention of consumers. Moisture loss, oil intake and morphological shrinkage due to heat and mass transfer was found responsible for color changes in fried tilapia skin [9]. Lightness (L*), which is the most important color index of fried food, decreased with frying time in all the three frying methods (Table 2). AF obtained the brightest color while CF and VF had lower L* values. This was because in CF and VF moisture volatility and the frying oil absorbed by the tilapia skin reduced the reflection of light. Moreover, frying oil absorption favored melanoidin pigment formation through Maillard reaction [17,18]. The redness a* value increased with frying time and temperature in all the samples, except for VF8BE which showed a significantly pale color compared to others. The b* value in BE was the highest in AF among the three frying conditions while VF8 had the lowest b* value. Generally, the increase in b* value implied a golden-like color caused by the Maillard reaction or caramelization of sugar during frying, which is preferred by consumers [18]. The BK offered lower levels of all the three color attributes than the BE under the same frying conditions (Table 1). 

**Figure 3:**
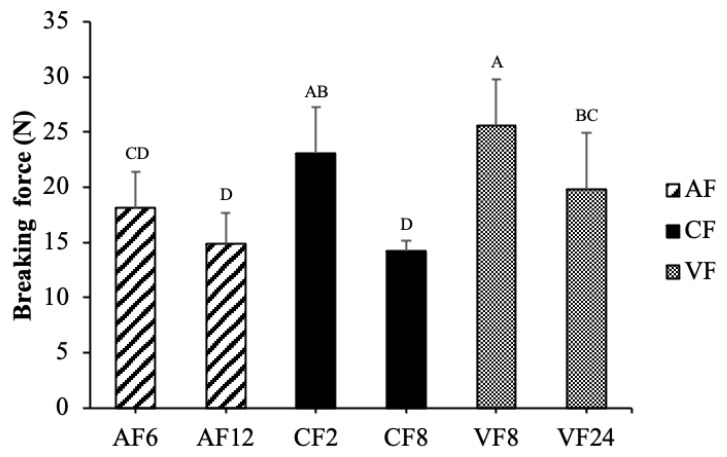
Changes in breaking force of fried tilapia skin with different frying methods. Different letter for any given treatment are significantly different (*n* = 3, *p* < 0.05). Abbreviation: AF6 & AF12 (Air frying at 6 and 12 min); CF2 & CF8 (Conventional frying at 2 and 8 min); VF8 & VF24 (Vacuum frying at 8 and 24 min).

Breaking force is an empirical determination of hardness and crispness, which is one of the most versatile quality properties of fried food. It decreased with frying time, which showed a positive correlation with moisture content, however, it was not influenced by the oil content of fried tilapia skin. Breaking force in CF showed an apparent reduction at 38% when time was extended to 8 min. Air frying seemed to develop a lower breaking force than CF and VF (Figure 3). It indicated the higher crispness of food cooked in AF, which might be due to the higher moisture loss and protein denaturation or the formation of shorter peptide chains [10,19].

### 2.2. Oil Content, Conjugated Diene Value (CDV) and TBARS

Oil content is a crucial feature in evaluating the quality of fried products and consumer preferences. CF had a higher oil absorption rate (31.09–42.27%, dry basis) among the three frying conditions (Table 2). VF showed a significantly lower fat content (17.24–22.18%) compared with CF. This was because VF fried tilapia skin was further processed by centrifuging at a lower temperature (120 °C) under vacuum, which reduced the oil absorption [9,17]. Air frying fried fish skin obtained the lowest oil content between 8.74 and 11.37%. Normally, fried food contains higher fat than its raw material. However, air fried fish skin offered lower oil content in the final product even when compared to the raw tilapia skin, which contained 16.45% fat (dry weight basis). This de-oiling ability was attributed to the fact that there was no extra addition of frying oil in AF, and the skin fat of tilapia was melted and removed by the circulated hot air with the increase in frying time. The oil content of CF fried Nile tilapia skin was 30.72% and AF was 14.68%, which is in alignment with our results [10]. AF foods were believed to have lower fat content and calories which adds to consumers’ health benefits [18]. 

Conjugated diene value (CDV) is a representative indicator for the primary lipid oxidation products caused by the double band shifts in PUFA during the frying process [20]. CDV significantly increased with frying time in the conventional frying method, especially in CF8 which reached the highest level at 5.62 and 6.02 mmol/g in BE and BK, respectively (Table 2). Vacuum frying seemed to have a lower CDV than CF and AF. In VF8BE, the lowest CDV level was observed at 0.97 mmol/g, which was slightly higher than the raw fish skin. It was found that BE exhibited lower CDV than BK under the same frying conditions (Table 2). This might be due to higher amount of fats (PUFAs), haemopigments, phospholipids, myoglobin and heme iron originated in the dark muscle of BK, which was regarded as a potent pro-oxidant in protein and lipid oxidation [21]. 

The thiobarbituric acid reactive substances (TBARS) represent the secondary oxidation products (aldehydes, and malonaldehyde (MDA)) of oil in the frying process [16]. CF groups did not show a significant change in TBARS after frying, and obtained the lowest level between 1.16 and 1.66 mg MDA/kg among all the frying conditions (Table 2). This was due to the migration of MDA from fried products to frying oils or due to the formation of MDA protein adducts [22]. Similar results were found in fried silver catfish, in which no significant differences in TBARS were observed between raw and fried samples [23]. The TBARS values of AF and VF were higher than the raw tilapia skin and showed a significant increasing trend with the addition of frying time and temperature. TBARS values of AF fried hairtail fillet were in agreement with our results [18]. In comparison to the TBARS in BK and BE under the same frying conditions, BK showed higher TBAS than BE; this might due to the fact that BK contained dark muscle which was susceptible to oxidation. AF12 obtained the highest value of 5.95 mg MDA/kg (Table 2). This might due to the concentration effect (moisture loss) and due to no oil working as a solvent to dissolve, dilute and remove TBARS.

### 2.3. Volatile Flavor Compounds

Aroma (volatile odor compounds, VOCs) was one of the major characteristics of fried food. It reflected the food quality and consumers’ attraction. The concentration of VOCs in AF12BK was the highest (4180 ppb) while it was the lowest in AF6BE (1169 ppb) (Table 3). The contents of VOCs significantly increased with increasing frying time. Although CF contained higher levels of VOCs than AF and VF, the diversity of VOCs in CF was the lowest (nine kinds); this was due to the presence of high amounts of (E,E)-2,4-decadienal and (E)-2-dodecenal in CF than the other two groups. The lowest VOCs in CF fried fish skin was in agreement with the previous report [10], and this might be because of less frying time, and potential migration of VOCs into frying oil. (E,E)-2,4-decadienal and (E)-2-dodecenal were the products resulting from the of oxidation and degradation of fatty acids and triglycerides in high temperature [21,24]. There were mainly 17 VOCs identified in fried fish skin via headspace solid-phase microextraction (HS-SPME) combined with GC-MS. The aldehydes, C5–C9, were found in large quantities in fried foods, which mainly came from frying oil [10,11,18]. Heterocyclic compounds, such as pyrazines, representing roasted and baked flavor were found more in AF, which indicated that the AF method might produce food with rich flavor. A total of 10 aldehydes were found in soybean oil-fried Nile tilapia meat, and they were regarded as the main odorants. Aldehydes generally offered low olfactory thresholds, and were generated at high quantities during thermal processing of fatty aquatic foods [11]. The most intensive VOCs (that obtained the highest flavor dilution factor) was (E,E)-2,4-decadienal with an oily odor defined by an aroma extract dilution analysis. The subsequent test proved that (E,E)-2,4-decadienal was one of nine contributing odorants to the smell of the fried tilapia in the omission experiment [11].

BK contained higher VOCs content than BE, regardless of the frying methods. This might be due to the higher amount of fat (PUFAs), haemopigments, phospholipid, myoglobin and heme iron originating in the dark muscle of BK [25]. Phospholipid (diacylglycerol), which is predominant in BK, was highly susceptible to lipid oxidation than neutral fat (triacylglycerol) found in the light muscle of BE. In addition, PUFAs were mainly esterified at the *sn*-2 position in marine phospholipid which caused high oxidation during frying [26]. Haemopigments, myoglobin as well as the released heme and iron cause long-term effects and are regarded as potent pro-oxidant for protein and lipid oxidation [21]. The higher lipid oxidation levels of BK in terms of TBARS and conjugated dienes (Table 2) agreed with the higher contents of VOCs in BK.

### 2.4. Principle Component Analysis (PCA)

**Figure 4:**
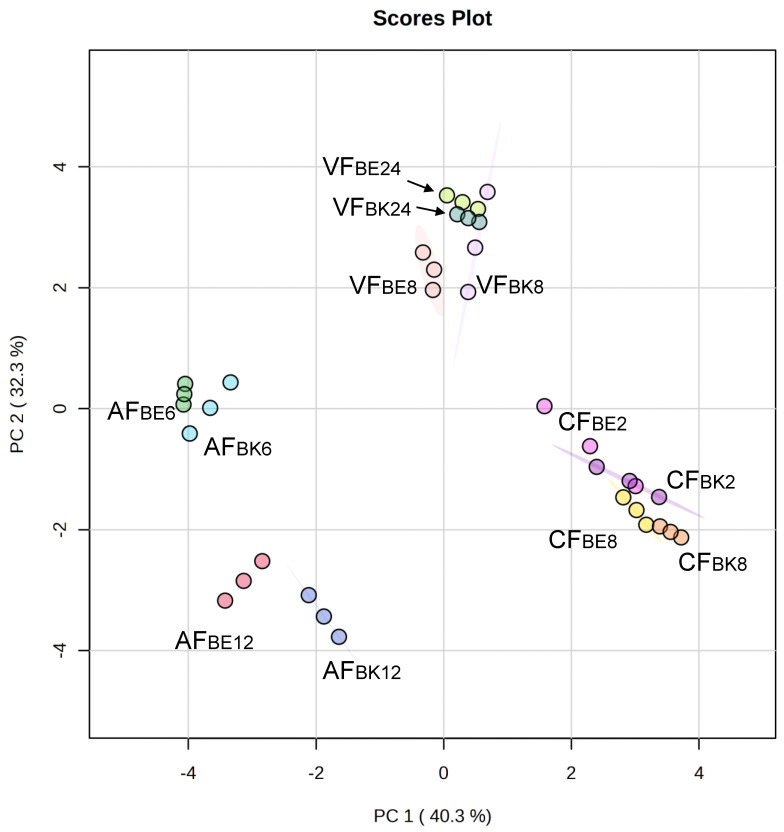
Principle component analysis (PCA) plot of the aroma attributes on different frying methods (AF, air frying; VF, vacuum frying; CF, conventional frying; BE, fish skin located in belly; BK fish skin located in back; the number indicated the frying time, *n* = 3).

The data of volatiles (Table 3) among different frying conditions were processed via principle component analysis (PCA) and are presented in Figure 4. The first two principal components (PCs) explained 73% of the total variation. PC1 separated AF, VF, and CF. Frying methods of AF and CF showed opposite response patterns. PC1 can be assigned as an attribute of frying method. Comparing the fried fish skin of near belly (BE) and back (BK) versus frying time, conventional frying method showed no difference on BE2, BK2, BE8, and BK8. This indicated that the aroma of fried skin by conventional frying mainly came from the frying oil. The frying oil was continuously heated and as such the aroma, characteristic of the fried fish skin observed, was irrelevant to the locations of fish skin cuts and the frying time. The oil content of CF8 fried tilapia skin increased from 16.45% in the raw material to 42.27%. Fried oil, as a heat transfer medium in CF and VF, under intensive heating could generate hydrogen peroxide and is further decomposed into large amounts of volatile aldehyde-derived compounds through thermal hydrolysis and degradation [11]. In VF and AF, frying time significantly influenced the aroma generation than the locations of fish skin (BE and BK). The clusters of fried fish skins in the same frying time were closer than the same locations of fish skin cuts. The aroma of fried fish skin did not originate in the raw material, but it was generated after heating, also known as the so-called reaction flavor. The Maillard reaction and Strecker degradation-derived flavor development was more time- and temperature-dependent [27]. The AF method without the use of oil showed significant results on heating for 12 min (AFBE12, and AFBK12) and 6 min (AFBE6, and AFBK6). Because air frying method fried the fish skin using only hot air, the aroma generated found was mainly the Maillard reaction products, such as methylpyrazine, 2,5-dimethylpyrazine, and benzaldehyde. This made the aroma profiles of AF very different from VF and CF. 

**Figure 5:**
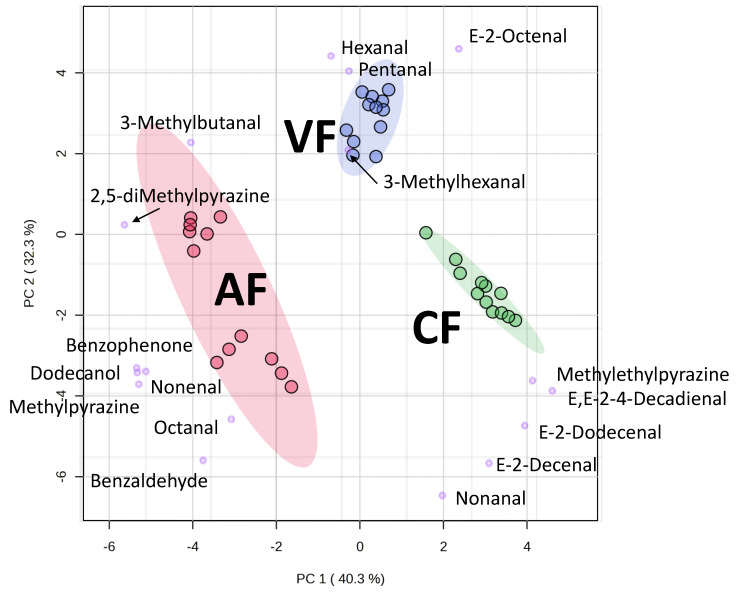
The biplot of principal component analysis (PCA) for the identified volatiles in fried tilapia skin by air frying, vacuum frying, and conventional frying methods (*n* = 3).

The loading score of each variable in the PCA was plotted along with the PC score to generate the biplot shown in Figure 5. The aroma compounds near the clusters of frying methods indicated their correlations to the frying methods. Therefore, it was observed that CF favored nonanal, decenal, and 2,4-decadienal, which were all lipid degradation products. The 2-decenal, 2,4-decadienal, and 2-undecenal were the main VOCs of oxidized linseed oil, sunflower oil, rapeseed oil and palm oil. They were decomposed to form intermediates, such as oleic acid 11-hydroperoxide, and linoleic acid 11-hydroperoxide [28]. In VF, pentanal, hexanal, 3-methylhexanal, and octenal were predominant. PC2 seemed to be associated with the temperature attribute. VF fried in lower temperature, hence, small aldehydes (C5–C7) were present. In addition, CF and AF fried in higher temperature, and thus large aldehydes (C9–C11) were observed. Moreover, all longer frying-time groups shifted further away from the origin in the score scattered plot. It implied that longer frying time provide more volatiles. 

## 3. Materials and Methods

### 3.1. Sample Preparation

Frozen skin of tilapia (*Oreochromis* spp.) was obtained from Fortune Life Enterprise Co., Ltd. (Kaohsiung City, Taiwan). Pieces of fish skin were cleaned after being scaled and frozen at −20 °C. Fish skin was thawed in running tap water, then cut into strips of about 5 cm × 3 cm. The surface water was wiped off prior to frying.

### 3.2. Frying Process

Fish skin was fried using the conventional deep-fat frying method [9] in a 1.2 L deep-fat fryer (KK-00458, D-Stylist, Japan). Refined palm oil (TTET Co., Ltd., Tainan City, Taiwan) was used and fish skins (10 pieces) were fried at 180 °C for 2 different intervals of time, 2 and 8 min. After frying, the fish skins were removed from the fryer and placed on a towel paper to eliminate the surface excess oil. A centrifugation device (deoiler DOO-130, Yu Sheng Guang Food Machine, Taichung, Taiwan) for de-oiling was then applied at 236 rpm for 3 min.

Air frying was carried out in a 2.2 L air fryer (HD 9642, Philips, Taiwan) at 180 °C. Fish skins (10 pieces) were placed in a wire basket and then air fried for 6 and 12 min. The air fryer was pre-heated at temperatures suitable for 4 min at the beginning of the experiments.

For vacuum frying, an industrial vacuum fryer (VF-5, Chin Ying Fa Mechanical Ind Co., Ltd., Chang Hua Hsien, Taiwan) was used. Eighteen litters of refined palm oil was preheated at 120 °C and under 0.085 MPa in vacuum. Fish skins (10 pieces) were fried for 8 and 24 min, followed by an automatic de-oiling process for 3 min (centrifugation).

### 3.3. Moisture Content, Water Activity, Color, and Breaking Force

Moisture content was determined using an infrared moisture meter as described in [17]. After calibration, 1 g of fried fish skin (cut to 2–3 mm) was placed in the sample plate for 1 h at 105 °C. The assay was performed in three repeats.

Water activity of fried tilapia skin was determined using a water activity meter (AQUALAB4TE, Meter Group, Inc., Pullman, WA, USA) [29]. One gram of fried fish skin (cut to 2–3 mm) was placed in the sample compartment for each measurement. The assay was performed in three repeats.

The color parameters, lightness (L*), redness (a*) and yellowness (b*) of fried fish skin was determined using a spectrocolorimeter (TC-1800 MK II, Tokyo, Japan) according to the method in [30]. Each mashed sample was measured in triplicate.

The maximum breaking force (N) of fried tilapia skin was determined via a modified method [13] using a texture analyzer (RapidTA, Horn Instruments Co., Ltd., Taoyuan, Taiwan). The samples were punctured with a spherical stainless-steel probe (P/0.25 s). The speed was set to 5.0 mm/s, and the trigger force was set at 10 g. The highest peak on the force–time curve was assumed as a hardness value. Each analysis was conducted 8 times.

### 3.4. Oil Content, TBARS, and Conjugated Diene Value (CDV)

Oil content of fried tilapia skin was determined gravimetrically after Soxhlet lipid extraction using a Teactor SoxtecSystem HT1043 (Foss Analytical Co., Ltd., Hillerod, Denmark) [9]. Dry weight basis (db) was determined by drying to a constant weight at 105 °C.

The thiobarbituric acid reactive substances (TBARS) value was examined according to the method in [31]. The sample (0.5 g) was mixed with a TBA reagent (0.25N HCl, 0.0375% thiobarbituric acid, and 15% TCA) and heated in a water bath at 100 °C for 10 min. The reference compound was 1,1,3,3-Tetramethoxypropane. The absorbance was measured at 532 nm and results were expressed as mg malonaldehyde/kg sample. 

Conjugated diene value (CDV) was measured with some modifications to [32]. Fish skin (cut to 2–3 mm) of 0.5 g was homogenized with an extracting solution (3:2 hexane:isopropanol) of 50 mL for 15 min. After centrifugation at 2000× *g* for 5 min, the absorbance of the supernatant was read at 233 nm. CDV was calculated using the molar extinction coefficient of 25,200 M^−1^ cm^−1^ and the results were expressed as mmol per g of sample.

### 3.5. Volatile Flavor Compound 

Aroma profile of fried fish skin was performed using the headspace solid phase micro extraction (HS-SPME) combined with gas chromatography mass spectrometry (GC-MS, Agilent 6890N GC, 5973 MSD). Separation was done via a DB-WAX capillary column. Sample 0.5 g each was placed in a 12 mL headspace vial, 4 mL of saturated NaCl solution, and internal standard (0.25 ppm of cinnamaldehyde) were added. The headspace vial was incubated and balanced at 70 °C for 10 min. The following adsorption was conducted via a polydimethylsiloxane (PDMS) fiber at 70 °C for 20 min. Volatiles were desorbed at 225 °C for 5 min via a GC injector. Helium was used as the carrier gas at a flow rate of 1 mL·min^−1^. The oven temperature was programed to 40 °C (held for 1 min), ramp 40–225 °C at 10 °C/min, 225 °C held for 15 min. The MS scanning range was set to m/z 33–450. The electron ionization was at 70 eV [33]. The identification of volatile compounds was carried out using matched standards and NIST library 5.0 (Gaithersburg, MD, USA).

### 3.6. Statistical Analysis

All data were expressed as mean ± SD and analyzed using one-way analysis of variance (ANOVA) which was performed via the General Linear Model (GLM) in the Statistical Products & Services Solution 20 (SPSS20) statistics software package (SPSS Inc., Chicago, IL, USA). Differences between means were evaluated via Ducan’s Multiple Range. The means bearing, unlike letters, differed significantly (*p* < 0.05). Linear correlations were tested using Pearson correlation analysis at *p* < 0.01 significance level. Principal component analysis (PCA) was performed in Metaboanalyst 5.0. (PCA), which was applied to explore the effects of different frying parameters toward aroma compounds (variables). The semi-quantitated area of each volatile was mean-centered and divided by the standard deviation (Z-score).

## 4. Conclusions

Fish skin is a by-product of aquaculture industries. Using fish skin to make fried fish skin products is a sustainable environmental activity, which would be much more beneficial if aquaculture industries can also take the health issues into account when recycling the by-products. Thus, low-fat fried fish skin products will be valued by consumers and food processors in the market. This study investigated the volatile and physiochemical properties of fried fish skins under various frying methods including air frying, vacuum frying, and deep-fat frying. Conventional fried tilapia skin had higher oil absorption (31.09–42.27%) rate among the three frying conditions. Vacuum frying showed a significantly lower oil content (17.24–22.18%) compared with conventional frying. Air frying fried fish skin, without the addition of oil, obtained the lowest oil contents between 8.74 and 11.37%. It also offered lower oil content in the final product even when compared to the raw tilapia skin, which contained 16.45% fat. The fried fish skins in oil frying obtained similar flavor profiles due to the transfer of most of the detectable aroma compounds from the frying oils into the food. Air frying offered a characteristic flavor distinct from vacuum and deep-fat frying because there was no oil used in the frying process. The aliphatic aldehydes, mainly C9 to C11, dominated the flavor of oil fried fish skins and gave a familiar taste of other oil fried foods. However, air frying not only offered aliphatic aldehydes, but also provided higher Maillard reaction-derived flavor notes, such as pyrazines and benzaldehyde. Vacuum frying offered low-fat fried fish skin with the flavor note similar to conventional deep-fat frying product. Air frying was able to keep the original flavor and add special flavor, especially a roasted flavor note to the product. It is concluded that vacuum and air frying both can be practically applied for cooking fried fish skin with low fat. 

## Figures and Tables

**Table 1:** Changes in color value of fried tilapia skin under various frying methods and parameters.

	AF6	AF12	CF2	CF8	VF8	VF24
			L* value			
BE	58.93 ± 2.55 ^Ac^	54.13 ± 3.68 ^ABb^	49.71 ± 2.74 ^BCa^	44.13 ± 5.80 ^Db^	54.79 ± 2.46 ^Aba^	44.88 ± 0.58 ^CDb^
BK	45.28 ± 1.48 ^Ac^	40.94 ± 2.36 ^Bd^	40.49 ± 3.14 ^Bbc^	37.66 ± 1.03 ^Cc^	42.28 ± 0.69 ^Bc^	36.75 ± 0.28 ^Cd^
			a* value			
BE	−3.38 ± 0.91 ^Aa^	−3.21 ± 0.86 ^Aa^	−3.70 ± 1.58 ^Aab^	−2.64 ± 2.61 ^Aa^	−8.58 ± 0.95 ^Bb^	−4.91 ± 0.12 ^Aa^
BK	−5.50 ± 0.90 ^ABb^	−5.37 ± 0.81 ^Ab^	−6.41 ± 0.36 ^Bc^	−4.85 ± 0.82 ^Abc^	−7.88 ± 0.04 ^Cb^	−5.26 ± 0.25 ^Aa^
			b* value			
BE	39.65 ± 1.87 ^Aa^	40.49 ± 1.35 ^Aa^	30.90 ± 3.47 ^Cb^	35.35 ± 1.56 ^Ba^	25.23 ± 3.04 ^Db^	30.60 ± 0.67 ^Ca^
BK	26.22 ± 5.08 ^Ab^	27.55 ± 1.35 ^Ab^	23.95 ± 2.57 ^ABc^	28.11 ± 2.86 ^Ab^	21.47 ± 0.30 ^Bc^	21.48 ± 0.54 ^Bc^

Different uppercase letter for any given treatment and row are significantly different (*p* < 0.05). Different lowercase letter for any given frying methods are significantly different (*p* < 0.05). Expressed as mean ± standard deviation (*n* = 3). Abbreviation: AF6 & AF12 (Air frying at 6 and 12 min); CF2 & CF8 (Conventional frying at 2 and 8 min); VF8 & VF24 (Vacuum frying at 8 and 24 min); BE (Fish skin on the belly); BK (Fish skin on the back).

**Table 2:** Oil contents and lipid oxidation levels of various fried tilapia skins.

	Raw	AF6	AF12	CF2	CF8	VF8	VF24
Crude fat (dry basis, %)							
	16.45 ± 0.69 ^D^	11.37 ± 1.49 ^E^	8.74 ± 2.65 ^E^	31.09 ± 3.33 ^B^	42.27 ± 2.23 ^A^	17.24 ± 1.82 ^D^	22.18 ± 1.39 ^C^
Conjugated diene value (CDV, mmol/g)							
BE	0.54 ± 0.03 ^Eb^	1.31 ± 0.31 ^Dc^	2.32 ± 0.37 ^Cb^	3.16 ± 0.25 ^Bb^	5.62 ± 0.40 ^Aa^	0.97 ± 0.22 ^DEc^	2.40 ± 0.47 ^Ca^
BK	0.84 ± 0.12 ^Fa^	1.56 ± 0.33 ^Ec^	4.30 ± 0.27 ^Ba^	3.69 ± 0.17 ^Cb^	6.02 ± 0.65 ^Aa^	1.48 ± 0.11 ^Eb^	2.11 ± 0.08 ^Da^
TBARS (mg MDA/kg)							
BE	1.09 ± 0.09 ^Da^	2.84 ± 0.41 ^Cd^	4.92 ± 0.35 ^Ab^	1.16 ± 0.07 ^Dc^	1.48 ± 0.06 ^Dab^	2.83 ± 0.45 ^Cc^	3.44 ± 0.12 ^Bab^
BK	1.50 ± 0.29 ^Da^	3.83 ± 0.42 ^Bc^	5.95 ± 0.28 ^Aa^	1.30 ± 0.08 ^Dbc^	1.66 ± 0.23 ^Da^	2.92 ± 0.26 ^Cbc^	3.93 ± 0.26 ^Ba^

Different uppercase letter for any given treatment and row are significantly different (*p* < 0.05). Different lowercase letter for any given frying methods are significantly different (*p* < 0.05). Expressed as mean ± standard deviation (*n* = 3). Abbreviation: AF6 & AF12 (Air frying at 6 and 12 min); CF2 & CF8 (Conventional frying at 2 and 8 min); VF8 & VF24 (Vacuum frying at 8 and 24 min); BE (Fish skin on the belly); BK (Fish skin on the back).

**Table 3:** Volatile profiles of fried tilapia skins under different frying methods.

Relative Content (ppb)	AF6		AF12		CF2		CF8		VF8		VF24	
	BE	BK	BE	BK	BE	BK	BE	BK	BE	BK	BE	BK
Pentanal	n.d.	n.d.	23 ± 3	16 ± 4	n.d.	n.d.	n.d.	n.d.	n.d.	90 ± 53	125 ± 25	120 ± 13
Hexanal	64 ± 9	128 ± 3	41 ± 9	198 ± 7	66 ± 16	80 ± 33	59 ± 19	40 ± 13	271 ± 1	235 ± 23	433 ± 68	403 ± 128
Octanal	n.d.	n.d.	96 ± 19	86 ± 4	n.d.	n.d.	n.d.	n.d.	8 ± 5	18 ± 0	n.d.	n.d.
Nonanal	1 ± 0	304 ± 31	679 ± 59	730 ± 177	495 ± 68	651 ± 106	793 ± 53	736 ± 64	113 ± 13	115 ± 35	170 ± 48	158 ± 98
3-Methylbutanal	125 ± 13	98 ± 15	n.d.	n.d.	n.d.	n.d.	n.d.	n.d.	63 ± 28	n.d.	63 ± 38	55 ± 30
3-Methylhexanal	n.d.	n.d.	n.d.	n.d.	n.d.	n.d.	n.d.	n.d.	25 ± 18	29 ± 20	n.d.	n.d.
(*E*)-2-Octenal	n.d.	n.d.	n.d.	n.d.	65 ± 13	80 ± 23	96 ± 11	101 ± 19	141 ± 1	168 ± 48	233 ± 8	205 ± 18
(*E*)-2-Nonenal	166 ± 6	210 ± 30	280 ± 38	309 ± 76	n.d.	n.d.	n.d.	n.d.	44 ± 4	51 ± 16	68 ± 10	65 ± 0
(*E*)-2-Decenal	n.d.	n.d.	346 ± 39	475 ± 160	320 ± 75	419 ± 64	526 ± 41	545 ± 30	85 ± 5	114 ± 26	148 ± 0	145 ± 15
(*E*)-2-Dodecenal	68 ± 15	105 ± 8	341 ± 26	710 ± 8	550 ± 170	711 ± 99	739 ± 51	819 ± 29	226 ± 16	269 ± 56	320 ± 10	340 ± 15
(*E*,*E*)-2,4-Decadienal	75 ± 8	126 ± 4	565 ± 43	929 ± 259	1015 ± 43	1400 ± 75	1451 ± 59	1659 ± 64	424 ± 11	540 ± 105	645 ± 108	623 ± 90
Benzaldehyde	244 ± 36	230 ± 53	211 ± 24	365 ± 85	66 ± 21	68 ± 5	129 ± 14	146 ± 9	19 ± 6	15 ± 3	20 ± 5	23 ± 10
1-Dodecanol	60 ± 23	64 ± 36	78 ± 26	45 ± 13	n.d.	n.d.	n.d.	n.d.	n.d.	n.d.	n.d.	n.d.
Methylpyrazine	45 ± 13	35 ± 3	44 ± 16	55 ± 8	n.d.	3 ± 1	3 ± 1	n.d.	21 ± 4	n.d.	n.d.	n.d.
Methylethenylpyrazine	n.d.	n.d.	n.d.	n.d.	9 ± 6	9 ± 4	8 ± 0	13 ± 0	n.d.	n.d.	n.d.	n.d.
2,5-Dimethylpyrazine	301 ± 1	374 ± 59	351 ± 99	251 ± 94	n.d.	n.d.	n.d.	n.d.	120 ± 45	179 ± 66	275 ± 25	228 ± 25
Benzophenone	20 ± 10	15 ± 5	21 ± 1	11 ± 1	n.d.	n.d.	n.d.	n.d.	n.d.	n.d.	n.d.	n.d.

Semi-quantitated by comparing the compound area to the internal standard 3-heptanone. n.d. means not detected. Expressed as mean ± standard deviation (*n* = 3). Abbreviation: AF6 & AF12 (Air frying at 6 and 12 min); CF2 & CF8 (Conventional frying at 2 and 8 min); VF8 & VF24 (Vacuum frying at 8 and 24 min); BE (Fish skin on the belly); BK (Fish skin on the back).
